# Supplementary material for: Early Archaean subduction and intracrustal processes: experimental evidence from the East Pilbara Terrane, Australia
Source: Nat Commun. 2026 Apr 4;17:4875. doi: 10.1038/s41467-026-71442-8 (PMC13230839; doi:10.1038/s41467-026-71442-8)
Supplement: Supplementary file 1 — Supplementary Information [file 41467_2026_71442_MOESM1_ESM.pdf]

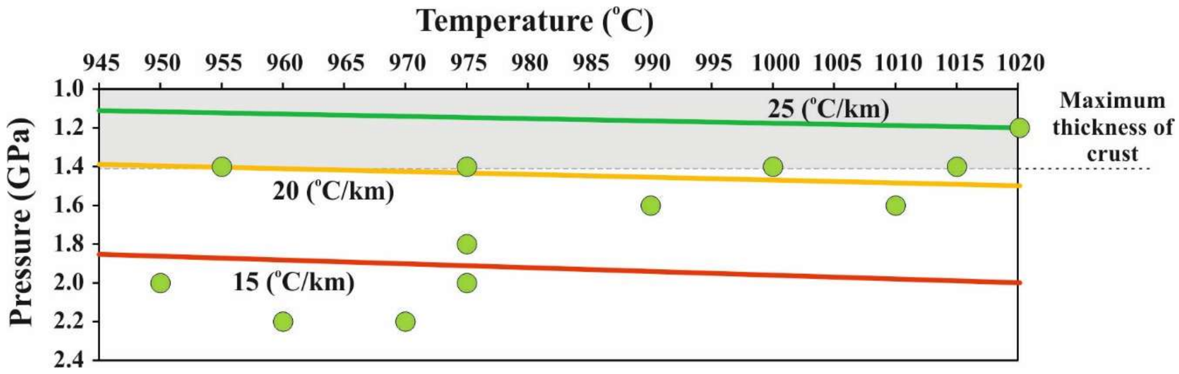

Fig. S1. *Diagrammatic cross section through the Eoarchaean-Palaeoarchaean Earth, showing 25, 20 and 15°C/km early Earth geothermal gradients and the P-T conditions of our experimental runs.*

	**SiO_2**	**TiO_2**	**Al_2O_3**	**FeO**	**MnO**	**MgO**	**CaO**	**Na_2O**	**K_2O**	**P_2O_5**
**Original 179789**	**47.76**	**2.06**	**15.39**	**14.19**	**0.25**	**7.42**	**9.50**	**2.36**	**0.94**	**0.14**
**EPT2019**	48.07	2.17	14.85	13.75	0.24	7.72	9.68	2.42	0.96	0.14
**EPT2021**	47.82	2.14	14.64	14.44	0.24	7.66	9.59	2.38	0.94	0.14
**EPT2024**	48.10	2.07	15.58	13.84	0.25	7.59	9.17	2.32	0.95	0.13
**1187-8**	49.22	0.75	14.87	9.76	0.17	9.89	12.39	1.64	0.09	0.06
**1187-10**	49.89	0.73	14.53	9.85	0.16	10.20	12.77	1.73	0.10	0.06

Table S1: *Comparison of starting material compositions*

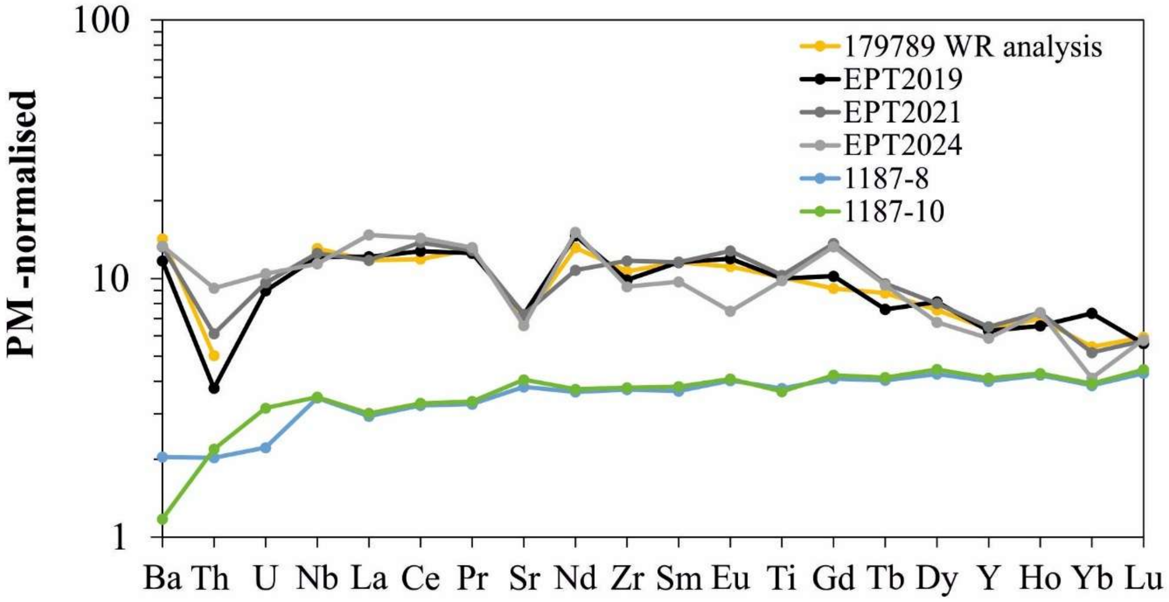

Figure S2: *Comparison of starting material trace element compositions for EPT2019, EPT2021 and EPT2024. Published whole rock (WR) 179789 analysis from Johnson et al.*[7]*. Previous oceanic plateau starting materials from Hastie et al.*[23,56] *(1187-8 and 1187-10) were originally analysed by Fitton and Godard*[57]*.*

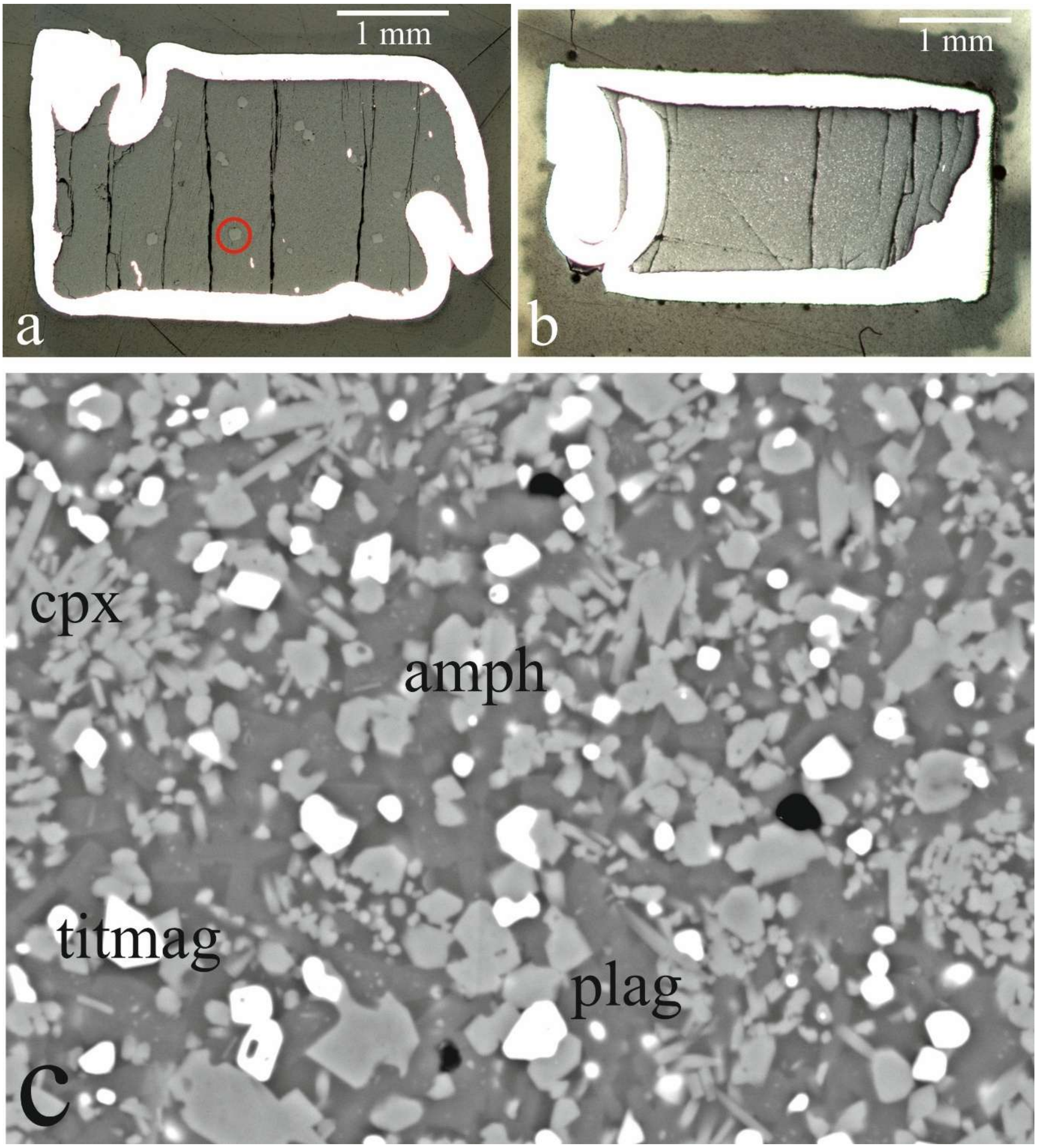

Figure S3: *Comparison of (a) garnet-bearing experiment EPTgw9 at 1.4 GPa (garnet example seen in red circle) and (b) experimental run EPTgw20 with no garnet at 1.2 GPa. (c) is an SEM image showing the amphibole, clinopyroxene (cpx), plagioclase (plag) and titanomagnetite (titmag) mineralogy of EPTgw20.*
